# Multi-Maneuvering Target Tracking Based on a Gaussian Process

**DOI:** 10.3390/s24227270

**Published:** 2024-11-14

**Authors:** Ziwen Zhao, Hui Chen

**Affiliations:** School of Electrical and Information Engineering, Lanzhou University of Technology, Lanzhou 730050, China; ziw@lut.edu.cn

**Keywords:** data-driven, multi-maneuvering target tracking, Gaussian process, model-free tracking

## Abstract

Aiming at the uncertainty of target motion and observation models in multi-maneuvering target tracking (MMTT), this study presents an innovative data-driven approach based on a Gaussian process (GP). Traditional multi-model (MM) methods rely on a predefined set of motion models to describe target maneuvering. However, these methods are limited by the finite number of available models, making them unsuitable for handling highly complex and dynamic real-world scenarios, which, in turn, restricts the adaptability and flexibility of the filter. In addition, traditional methods often assume that observation models follow ideal linear or simple nonlinear relationships. However, these assumptions may be biased in actual application and so lead to degradation in tracking performance. To overcome these limitations, this study presents a learning-based algorithm-leveraging GP. This non-parametric GP approach enables learning an unlimited range of target motion and observation models, effectively mitigating the problems of model overload and mismatch. This improves the algorithm’s adaptability in complex environments. When the motion and observation models of multiple targets are unknown, the learned models are incorporated into the cubature Kalman probability hypothesis density (PHD) filter to achieve an accurate MMTT estimate. Our simulation results show that the presented approach delivers high-precision tracking of complex multi-maneuvering target scenarios, validating its effectiveness in addressing model uncertainty.

## 1. Introduction

Maneuvering target tracking (MTT) involves monitoring the velocity, acceleration, position, and other state information of a moving target by using sensors that predict and track the target’s trajectory using algorithms. This technology has extensive applications in video surveillance, robotic vision, and military operations [1,2,3,4,5,6]. MTT remains challenging due to external environment and disturbance effects, where the target motion may exhibit irregular and highly dynamic characteristics [7].

Traditional MTT methods, which are model-driven (MD), describe the dynamic characteristics of the target through reasonable assumptions and modeling of target motion. These methods utilize recursive filtering techniques to process sensor measurements and system noise. The interactive multiple model (IMM) algorithm is a typical representative of this category. It employs multiple motion models simultaneously to describe different target motion models. It dynamically adjusts the weights of each model during filtering to achieve optimal state estimation of the target [8,9,10,11,12,13]. To further improve the flexibility and adaptability of MTT, ref. [14] proposed the variable-structure IMM algorithm, which handles changes in target motion models more effectively. However, these methods are primarily designed for single-target tracking problems. With an increasing number of targets in the surveillance area, the applicability of these methods decreases significantly.

The multi-model (MM) approach is an effective solution for multi-maneuvering target tracking (MMTT), which is widely applied to solving multi-target tracking problems with various motion patterns. Several MMTT filtering algorithms have been developed, based on this approach. For example, refs. [15,16] introduced the MM probability hypothesis density (PHD) filter; ref. [17] proposed an MM cardinalized PHD filter, to address the problem of inaccurate target cardinality estimation in MMTT; ref. [18] proposed a variable-structure MM-PHD (VSMM-PHD) filter to improve the efficiency and accuracy of MMTT. Unlike the traditional MM-PHD filter, VSMM-PHD uses a different set of models for each target at different times, better adapting to changes in target motion. In addition, refs. [19,20,21] developed various MM MeMBer filters to meet the needs of different tracking scenarios. However, as the uncertainty of target trajectories increases and the diversity of target motion patterns increases, model-based methods become increasingly inadequate for handling such complex variations. These methods are subject to certain limitations in practical application. Firstly, model-based methods rely heavily on initial conditions. Inaccurate initial settings can adversely affect estimation performance. Secondly, although increasing the number of models can improve tracking accuracy, an excessive number of models significantly increases the computational cost and complexity.

To overcome the limitations of the traditional methods, the data-driven (DD) approach, which is mainly based on a Gaussian process (GP) [22], provides promising alternatives. By contrast, GP-based techniques can learn the underlying models and model parameters from training data through non-parametric regression, thus eliminating the dependence on motion models in the classic MTT approach. The advantage of this strategy is its ability to adapt to different target motion models and produce more reliable state estimates. In recent years, GP-based target-tracking methods have increasingly become a popular research area, and they provide a substitute for traditional methods. A GP is a non-parametric machine learning regression method based on Bayesian inference. The distribution of output variables is modeled through a GP, which updates this distribution, using observational data. As a flexible model, a GP can adapt to various input and output data in multi-dimensional spaces and perform adaptive optimization based on the data. Moreover, a GP can seamlessly integrate state space models and Bayesian filtering. For instance, refs. [23,24] demonstrates using the GP to learn prediction and observation models from training data; ref. [25] combines Kalman filtering (KF) with a GP to create an efficient GP estimator for a spatiotemporal dynamic GP. Furthermore, ref. [26] modeled unknown perturbations as the GP and proposed an adaptive KF to improve the estimation performance.

Recent studies have applied the GP to MTT to address issues related to unknown target motion models or mismatches between motion models and actual target motion. For example, ref. [27] proposed a model-free MTT method that leverages the flexibility of the GP to enable switching between a large number of models and state estimates. Another study [28] introduced a DD method for MTT and smoothing, which showed significant performance improvements compared to traditional MD methods; ref. [29] presented a new GP-based approach to learning motion models and applied it within particle filtering to track targets in different surveillance regions. Furthermore, ref. [30] used a GP to approximate the transition density of the Bayesian optimal Bernoulli filter and proposed a particle implementation of the Bernoulli filter to handle unknown target motion model transitions, while [31] proposed a hybrid strategy that combines DD and MD approaches and effectively improves the tracking performance of strong maneuverable targets by integrating the advantages of both methods. Despite the success of these GP-based approaches in a variety of application scenarios, research for model-free MMTT in the context of random finite set (RFS) theory [32] has not yet been implemented. Further exploration of this area is essential to advance the development of MMTT technology.

To this end, this study proposes a novel MMTT algorithm to improve tracking accuracy in complex environments. The main contributions of this paper are as follows:

(1) A data-driven MMTT state estimation method is proposed by combining a GP and the PHD filter. The method models the MMTT motion and observation models as nonlinear functions over time. It uses a GP to learn the unknown characteristics of the target motion and observation models from training data.
(2) Based on the GP model learning, a cubature Kalman filter (CKF) [33] is utilized to propagate the uncertainty of the system to achieve accurate estimation. The GP possess provide model-learning capability, while the CKF efficiently handles nonlinear system through the ‘cubature sampling’ technique. This innovative design allows the GP-PHD filter to achieve excellent tracking accuracy and stability in uncertain and complex environments.
(3) To verify the effectiveness of the proposed algorithm, two groups of simulation experiments with different scenarios are designed. The results demonstrate that, compared to the traditional MD method, the GP-based method offers significant advantages in an environment with unpredictable and highly dynamic target motion.

Furthermore, the existing GP-based MTT algorithms are limited to scenarios involving a single target. However, the proposed algorithm overcomes this limitation, enabling simultaneous tracking of multi-maneuvering targets. This capability is particularly important in scenarios with numerous targets and frequent dynamic changes. The proposed method imposes no restrictions on the number of targets. It can effectively handle target generation, disappearance and maneuvering behavior, demonstrating its applicability and flexibility in complex MMTT scenarios.

The remainder of the paper is organized as follows. Section 2 introduces the problem definition and background, and Section 3 introduces the Gaussian process. A detailed implementation of the proposed algorithm is given in Section 4. Simulation results are provided in Section 5, and Section 6 concludes the paper.

## 2. Problem Definition and Background

### 2.1. System Model

Consider a discrete-time dynamic model with a transfer dynamics equation and observation equation as

(1) $$x_{t+1}=f\left(x_{t}\right)+\phi_{t}$$

(2) $$z_{t}=g\left(x_{t}\right)+\varsigma_{t}$$

where $x_{t}={\left[\zeta_{t},{\dot{\zeta }}_{t},\varphi_{t},{\dot{\varphi }}_{t}\right]}^{T}$ represents the state for a target in two-dimensional space at time *t*, $\left(\zeta_{t},{\dot{\zeta }}_{t}\right)$ represents the position along the *x*- and *y*-axis, $\left(\varphi_{t},{\dot{\varphi }}_{t}\right)$ denotes the corresponding velocity; $z_{t}$ denotes sensor measurement. The *f* and *g* are nonlinear process transfer functions and observation functions; $\phi_{t}$ and $\varsigma_{t}$ are the zero mean, white additive Gaussian process and measurement noise, respectively.

### 2.2. Multi-Target Bayesian Filtering

Based on the RFS theory [32], the state and measurement sets for multiple targets are represented as RFS $X_{t}=\left\{x_{t,1},\ldots ,x_{t,n_{x}}\right\}$ and $Z_{t}=\left\{z_{t,1},\ldots ,z_{t,n_{z}}\right\}$, respectively; $n_{x}$ and $n_{z}$ specify the number for targets and measurements, respectively. According to the Chapman–Kolmogorov Equation [34], the multi-target prediction equation at time *t* can be derived as

(3) $$\begin{array}{c} f_{t\left|t-1\right.}\left(X_{t}\left|Z_{1:t-1}\right.\right)=\int f_{t\left|t-1\right.}\left(X_{t}\left|X_{t\left|t-1\right.}\right.\right)f_{t-1}\left(X_{t\left|t-1\right.}\left|Z_{1:t-1}\right.\right)\delta X_{t\left|t-1\right.} \end{array}$$

where $f_{t\left|t-1\right.}$ and $f_{t-1}\left(X_{t\left|t-1\right.}\left|Z_{1:t-1}\right.\right)$ denote a multi-target state transfer function and state at time t−1, respectively. According to Bayes’ rule, after a new set $Z_{t}$ of measurements is received at time *t*, the multi-target update equation is given by

(4) $$\begin{array}{c} f_{t\left|t\right.}\left(X_{t\left|t\right.}\left|Z_{1:t}\right.\right)=\frac{L_{t\left|t\right.}\left(Z_{t\left|t\right.}\left|X_{t\left|t\right.}\right.\right)f_{t\left|t-1\right.}\left(X_{t\left|t\right.}\left|Z_{1:t-1}\right.\right)}{\int L_{t\left|t\right.}\left(Z_{t\left|t\right.}\left|X_{t\left|t\right.}\right.\right)f_{t\left|t-1\right.}\left(X_{t\left|t\right.}\left|Z_{1:t-1}\right.\right)\delta X_{t\left|t\right.}} \end{array}$$

### 2.3. PHD Filter

Suppose $\nu_{t}$ and $\nu_{t\left|t-1\right.}$ denote the intensity functions corresponding to multi-target posterior density $p_{t}$ alongside predicted density $p_{t\left|t-1\right.}$, respectively. The multi-target intensity function at time t−1 is given by the $\nu_{t}$, and its prediction equation of the PHD filter can be expressed as

(5) $$\begin{array}{c} \nu_{t\left|t-1\right.}\left(x\right)=\int p_{s,t}\left(x^{\prime }\right)f_{t\left|t-1\right.}\left(x\left|x^{\prime }\right.\right)\nu_{t-1}\left(x^{\prime }\right)dx^{\prime }+\int \beta_{t\left|t-1\right.}\left(x\left|x^{\prime }\right.\right)\nu_{t-1}\left(x^{\prime }\right)dx^{\prime }+\gamma_{t}\left(x\right) \end{array}$$

where $p_{s,t}\left(x^{\prime }\right)$ denotes the probability of surviving, $f_{t\left|t-1\right.}\left(x\left|x^{\prime }\right.\right)$ represents the transition probability density of a single target. At time *t*, $\beta_{t\left|t-1\right.}\left(x\left|x^{\prime }\right.\right)$ and $\gamma_{t}\left(x\right)$ represent the intensity of the spawned and birth targets, respectively. Given the set of measurements $Z_{t}$ at time *t*, the update Equation for the PHD filter is

(6) $$\begin{array}{c} \nu_{t\left|t\right.}\left(x\right)=\left(1-p_{d,t}\left(x\right)\right)\nu_{t\left|t-1\right.}\left(x\right)+\sum_{z\in Z_{t}}\frac{p_{d,t}\left(x\right)g_{t}\left(z\left|x\right.\right)\nu_{t\left|t-1\right.}\left(x\right)}{\kappa_{t}\left(z\right)+\int p_{d,t}\left(x^{\prime }\right)g_{t}\left(z\left|x^{\prime }\right.\right)\nu_{t\left|t-1\right.}\left(x^{\prime }\right)dx^{\prime }} \end{array}$$

where $p_{d,t}\left(x\right)$ denotes detection probability, $g_{t}\left(z\left|x\right.\right)$ represents the measurement likelihood of a single target, and $\kappa_{t}\left(z\right)$ signifies the intensity for clutter.

## 3. Gaussian Process

Using a training dataset, the GP is a complex non-parametric learning algorithm primarily used to learn unknown functions. The dataset contains input–output pairs, and the GP provides a mapping between them. The critical aspect that comprises GP involves the flexibility of modeling as it facilitates simulating the behavior of a system in the face of uncertainty.

### 3.1. Basic Gaussian Process Model

The GP represents a distribution of the function based on the training data. Suppose there is a set of training data $T_{d}=\langle X,y\rangle$, where *d*-dimensional input vector $x_{i}$ are arranged in the matrix $X=\left[x_{1},x_{2},\ldots ,x_{n}\right]$, where *n* denotes the number of training points, and $y=\left[y_{1},y_{2},\ldots ,y_{n}\right]$ is the vector containing the scalar training output. Assume that the measurement values are derived from the noise process

(7) $$y_{i}=h\left(x_{i}\right)+\varepsilon$$

where ε is additive Gaussian white noise with zero mean and variance is $\sigma_{n}^{2}$. The Gaussian predictive distribution on the output $y_{*}$ for training data $T_{d}=\langle X,y\rangle$ and test inputs $x_{*}$, the mean and variance are specified by the GP, i.e.,

(8) $$GP_{m}\left(x_{*},T_{d}\right)=k_{*}^{T}K^{-1}y$$

(9) $$GP_{v}\left(x_{*},T_{d}\right)=k\left(x_{*},x_{*}\right)-k_{*}^{T}K^{-1}k_{*}$$

In this case, $k_{*}$ is a vector formed by the kernel values between the test input $x_{*}$ and the training input *X*, where *k* indicates the kernel function for the GP, and the training input values are represented by the n×n dimensional kernel matrix *K*, which means that, $k_{*}\left[i\right]=k\left(x_{*},x_{i}\right)$ and $K\left[i,j\right]=k\left(x_{i},x_{j}\right)$. It should be emphasized that process noise, both the correlation between the test input and the training data, influence the prediction uncertainty as reflected by variance $GP_{v}$.

**Figure 1:**
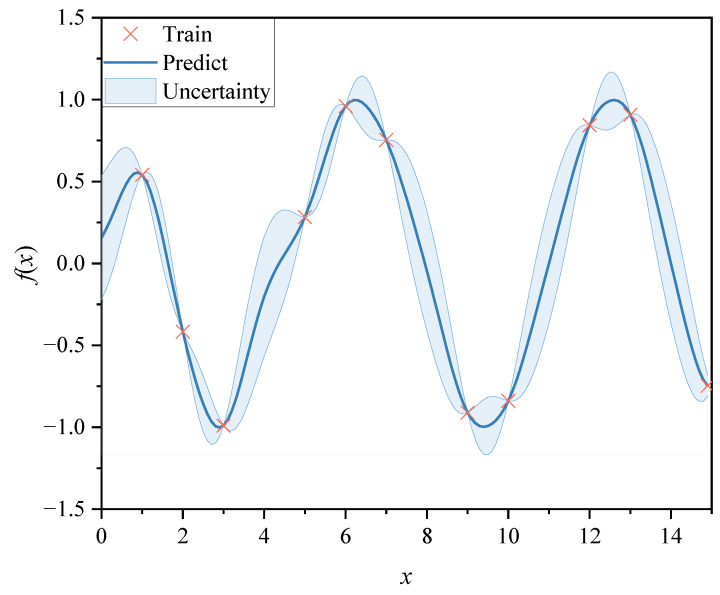
One-dimensional GP.

The exact application scenario determines the kernel function to be utilized, with the squared exponential or the Gaussian kernel with additive noise being the most popular

(10) $$k\left(x,x^{\prime }\right)=\sigma_{f}^{2}e^{-\frac{1}{2}\left(x-x^{\prime }\right)A{\left(x-x^{\prime }\right)}^{T}}+\sigma_{n}^{2}\delta$$

and the signal variance is given by $\sigma_{f}^{2}$, thus regulating the degree of prediction uncertainty in the area of low training data density. The length scale of the process is contained in the diagonal matrix *A*, for example, A=diag11l12a12,11l12a12,…,11ld2ad2. In different input dimensions, the length scale reacts to how smooth the operation is overall. $\sigma_{n}^{2}$ is the final GP parameter, which controls the noise of the whole process. Figure 1 illustrates a one-dimensional GP example. In the figure, the red × denotes the training point, the blue curve represents the prediction result, and the blue shading represents uncertainty. It can be seen from the figure that the uncertainty is lower near the training points and increases in areas away from the training points.

### 3.2. Hyperparameter Learning

The hyperparameters of the GP are represented by $\theta =\left[A,\sigma_{f},\sigma_{n}\right]$. By maximizing the log marginal likelihood of the training output for a given input, they can be trained

(11) $$\theta_{\max}=\underset{\theta }{\arg\max}\left\{\log\left(p\left(y\left|X,\theta \right.\right)\right)\right\}$$

It is possible to express the logarithmic component in (11) as

(12) $$\begin{array}{c} \log\left(p\left(y\left|X,\theta \right.\right)\right)=-\frac{1}{2}y^{T}{\left(K\left(X,X\right)+\sigma_{n}^{2}I\right)}^{-1}y-\frac{1}{2}\log\left|K\left(X,X\right)+\sigma_{n}^{2}I\right|-\frac{n}{2}\log2\pi \end{array}$$

Numerical optimization methods such as conjugate gradient ascent can be employed to solve this optimization problem [21]. To perform this optimization, it is essential to use the partial derivatives of the log-likelihood, as given below

(13) $$\frac{\partial }{\partial \theta_{t}}\log\left(p\left(y\left|X,\theta \right.\right)\right)=\frac{1}{2}tr\left[\left(K^{-1}y\right){\left(K^{-1}y\right)}^{T}\frac{\partial K}{\partial \theta_{t}}\right]$$

Each element of $\frac{\partial K\left[i,j\right]}{\partial \theta_{t}}$ in (13) represents a partial derivative of a kernel function in regard to its hyperparameters

(14) $$\frac{\partial k\left(x_{i},x_{j}\right)}{\partial \sigma_{f}}=2\sigma_{f}e^{-\frac{1}{2}\left(x_{i}-x_{j}\right)A{\left(x_{i}-x_{j}\right)}^{T}}$$

(15) $$\frac{\partial k\left(x_{i},x_{j}\right)}{\partial \sigma_{n}}=2\sigma_{n}\delta$$

(16) $$\frac{\partial k\left(x_{i},x_{j}\right)}{\partial A_{ii}}=-\frac{1}{2}{\left(x_{i}\left[i\right]-x_{j}\left[i\right]\right)}^{2}\sigma_{f}^{2}e^{-\frac{1}{2}\left(x_{i}-x_{j}\right)A{\left(x_{i}-x_{j}\right)}^{T}}$$

Due to the non-convex nature of this optimization problem, finding the global optimal solution cannot be guaranteed. However, in practical applications, such optimization problems often yield satisfactory results.

### 3.3. Learning Prediction and Observation Models Using Gaussian Process

The GP is possibly employed straight to the Bayesian filter in (3), and it has been shown to satisfy the conditions for learning predictive and observational models. In the context of the application in this work, the model needs to provide both expected mean and predicted uncertainty or noise. The GP inherently satisfies both objectives in its unique manner.

The training data are obtained by dynamically sampling and observing the system. They are expected to be representative of the system, i.e., they can span the state space encountered during normal operation. A set of input–output relations forms the training data for each GP. In the predictive model, state and control variables $\left(x_{t},u_{t}\right)$ are mapped to state transitions $\Delta x_{t}=x_{t+1}-x_{t}$. Then, the previous state is added to the state transition to determine the subsequent state. The state $x_{t}$ is mapped into observation $z_{t}$ using the observation model. Consequently, the training dataset for prediction and observation should have the following form

(17) $$T_{p}=\langle \left(X,u\right),X^{\prime }\rangle$$

(18) $$T_{o}=\langle X,Z\rangle$$

where the matrix containing the real states is indicated by *X*, and the matrix created when these states experience a transfer of control in application *u* is $X^{\prime }=\left[\Delta x_{1},\Delta x_{2},\ldots ,\Delta x_{t}\right]$. The observation matrix for the corresponding state *X* is denoted by *Z*. The prediction and observation models for the GP are subsequently obtained as

(19) $$\begin{array}{c} p\left(x_{t}\left|x_{t-1},u_{t-1}\right.\right)\approx N\left(GP_{m}\left(\left[x_{t-1},u_{t-1}\right],T_{p}\right),GP_{v}\left(\left[x_{t-1},u_{t-1}\right],T_{p}\right)\right) \end{array}$$

(20) $$p\left(z_{t}\left|x_{t}\right.\right)\approx N\left(GP_{m}\left(x_{t},T_{o}\right),GP_{v}\left(x_{t},T_{o}\right)\right)$$

It is important to note that the mean and variance of these models, for both input and training data, are nonlinear functions, even though they correspond to a Gaussian distribution. Moreover, due to their local Gaussian character, these models are seamlessly integrated into Bayesian filters.

The GP is typically defined in the case of scalar outputs. However, the GP Bayesian filter is represented for the vector output model by learning a distinct GP for each output dimension. Since the output dimensions are no longer interdependent, the resulting noise covariance matrix of the GP becomes diagonal.

## 4. Gaussian Process Bayesian Filter

In the following phase, a GP model will be introduced into the Bayesian filter to address the uncertainty in the motion and observation models of MMTT.

### 4.1. Gaussian Process for System Model

Some existing MD methods represent the motion and observation states of a target through one or more defined equations of motion and observations. However, GP-based approaches eliminate the need for precise equations of motion and observation. This reduces the reliance on the target motion and observation models by encoding the target state through the learned GP state and observation models.

The GP state model $GP^{f}$ and observation model $GP^{h}$ can be used to express the state and measurement equations as shown below

(21) $$x_{t}=GP_{m}^{f}\left(\left[x_{t-1},u_{t-1}\right],T_{p}\right)+\phi_{t-1}$$

(22) $$z_{t}=GP_{m}^{h}\left(x_{t},T_{o}\right)+\varsigma_{t}$$

where

(23) $$\phi_{t-1}∼N\left(0,GP_{v}^{f}\left(\left[x_{t-1},u_{t-1}\right],T_{p}\right)\right)$$

(24) $$\varsigma_{t}∼N\left(0,GP_{v}^{h}\left(x_{t},T_{o}\right)\right)$$

### 4.2. GP-CK-PHD Gaussian Mixture Implementation

Based on the Gaussian mixture (GM) recursive construction of the standard PHD filter, the posterior intensity of the multi-target state is expressed as a weighted sum of multiple non-Gaussian functions, derived through the recursive propagation in (5) and (6). Gaussian functions can approximate each non-Gaussian component, and similar to the CKF method, the ‘cubature sampling’ approach can be used to calculate the GM approximating components of the posterior intensity at subsequent time steps while approximating the weight of each component.

Therefore, this study proposes a nonlinear GM implementation based on the GP-PHD filter to address the challenges posed by uncertain motion and observation models in MMTT. This method leverages the GP learning approach and employs cubature sampling for propagation, making it an effective solution for tackling the problems of uncertain motion and observation models under nonlinear conditions in MMTT.

Considering the properties of nonlinear systems, it is impossible to represent the posterior intensity explicitly in GM form, so it is necessary to approximate the non-Gaussian component of the posterior intensity using an appropriate Gaussian distribution. The GM form for the birth RFS intensity is

(25) $$\gamma_{t}\left(x\right)=\sum_{a=1}^{J_{\gamma ,t}}w_{\gamma ,t}^{a}N\left(x;m_{\gamma ,t}^{a},P_{\gamma ,t}^{a}\right)$$

where $J_{\gamma ,t}$, $w_{\gamma ,t}^{a}$, $m_{\gamma ,t}^{a}$, $P_{\gamma ,t}^{a}$, $a=1,\ldots ,J_{\gamma ,t}$ are the model parameter given to determine the birth intensity. The particular procedure is described below:

(1) Consider the following as an approximation of the posterior intensity at time t−1 can be approximated by

(26) $$\nu_{t-1}\left(x\right)\approx \sum_{a=1}^{J_{t-1}}w_{t-1}^{a}N\left(x;m_{t-1}^{a},P_{t-1}^{a}\right)$$

Then, at the time *t*, the predicted intensity is

(27) $$\nu_{t\left|t\right.-1}\left(x\right)=\nu_{s,t\left|t\right.-1}\left(x\right)+\gamma_{t}\left(x\right)$$

where

(28) $$\nu_{s,t\left|t\right.-1}\left(x\right)\approx p_{s,t}\sum_{j=1}^{J_{t-1}}w_{t-1}^{j}N\left(x;m_{s,t\left|t-1\right.}^{j},P_{s,t\left|t-1\right.}^{j}\right)$$

According to the Cubature rule, 2n weighted Cubature sampling points $\left[x_{t\left|t-1\right.}^{l},w_{t\left|t-1\right.}^{l}\right]$ are selected, and the quantity of sampling points is l=0,1,…,2n. Then, the model of the unknown system is linearized, where

(29) $$x_{l,t-1}=x_{t-1}\pm \sqrt{P_{t-1}}\alpha_{l}$$

(30) $$x_{t\left|t-1\right.}^{l}=GP_{m}\left(\left[x_{l,t-1},u_{t-1}\right],T_{p}\right)$$

(31) $$Q_{t}=GP_{v}\left(\left[x_{t-1},u_{t-1}\right],T_{p}\right)$$

(32) $$m_{s,t\left|t-1\right.}^{j}=\frac{1}{2n}\sum_{l=0}^{2n}x_{t\left|t-1\right.}^{l}$$

(33) $$P_{s,t\left|t-1\right.}^{j}=\frac{1}{2n}\sum_{l=0}^{2n}\left(x_{t\left|t-1\right.}^{l}-m_{s,t\left|t-1\right.}^{j}\right){\left(x_{t\left|t-1\right.}^{l}-m_{s,t\left|t-1\right.}^{j}\right)}^{T}+Q_{t}$$

(2) Suppose that a Gaussian mixture can be used to roughly represent the predicted intensity at time *t*, i.e.,

(34) $$\nu_{t\left|t-1\right.}\left(x\right)\approx \sum_{j=1}^{J_{t\left|t-1\right.}}w_{t\left|t-1\right.}^{j}N\left(x;m_{t\left|t-1\right.}^{j},P_{t\left|t-1\right.}^{j}\right)$$

Then the posterior intensity at time *t* is likewise in the structure of a GM, denoted as

(35) $$\nu_{t}\left(x\right)=\left(1-p_{d,t}\right)\nu_{t\left|t-1\right.}\left(x\right)+\sum_{z\in Z_{t}}\nu_{d,t}\left(x;z\right)$$

where

(36) $$\nu_{d,t}\left(x;z\right)=\sum_{j=1}^{J_{t\left|t-1\right.}}w_{t}^{j}\left(z\right)N\left(x;m_{t\left|t\right.}^{j}\left(z\right);P_{t\left|t\right.}^{j}\right)$$

(37) $$w_{t}^{j}\left(z\right)=\frac{p_{d,t}w_{t\left|t-1\right.}^{j}q_{t}^{j}\left(z\right)}{\kappa_{t}\left(z\right)+p_{d,t}\sum_{j=1}^{J_{t\left|t-1\right.}}w_{t\left|t-1\right.}^{j}q_{t}^{j}\left(z\right)}$$

(38) $$w_{t\left|t-1\right.}^{j}=p_{s,t}w_{t-1}^{j}$$

(39) $$q_{t}^{j}\left(z\right)=N\left(z;\eta_{t\left|t-1\right.}^{j},S_{t}^{j}\right)$$

(40) $$m_{t\left|t\right.}^{j}\left(z\right)=m_{s,t\left|t-1\right.}^{j}+K_{t}^{j}\left(z-\eta_{t\left|t-1\right.}^{j}\right)$$

(41) $$x_{t\left|t\right.}^{l}=m_{s,t\left|t\right.-1}^{j}\pm \sqrt{P_{s,t\left|t\right.-1}^{j}}\alpha_{l}$$

(42) $$z_{t\left|t-1\right.}^{l}=GP_{m}\left(x_{t\left|t\right.}^{l},T_{o}\right),\;\;\;\;\;\;\;\;\;\;\;\;l=0,\ldots ,2n$$

(43) $$R_{t}=GP_{v}\left(m_{t\left|t-1\right.}^{j},T_{o}\right)$$

(44) $$\eta_{t\left|t-1\right.}^{j}=\frac{1}{2n}\sum_{l=0}^{2n}z_{t\left|t-1\right.}^{l}$$

(45) $$S_{t}^{j}=\frac{1}{2n}\sum_{l=0}^{2n}\left(z_{t\left|t-1\right.}^{l}-\eta_{t\left|t-1\right.}^{j}\right){\left(z_{t\left|t-1\right.}^{l}-\eta_{t\left|t-1\right.}^{j}\right)}^{T}+R_{t}$$

(46) $$P_{xz,t}^{j}=\frac{1}{2n}\sum_{l=0}^{2n}{\left(x_{t\left|t-1\right.}^{l}-m_{s,t\left|t-1\right.}^{j}\right)}^{T}{\left(z_{t\left|t-1\right.}^{l}-\eta_{t\left|t-1\right.}^{j}\right)}^{T}$$

(47) $$K_{t}^{j}=P_{xz,t}^{j}{\left(S_{t}^{j}\right)}^{-1}$$

(48) $$P_{t\left|t\right.}^{j}=P_{t\left|t-1\right.}^{j}-K_{t}^{j}S_{t}^{j}{\left(K_{t}^{j}\right)}^{-1}$$

Given the GM intensity $\nu_{t\left|t-1\right.}$ and $\nu_{t}$, the appropriate weights can be summed jointly to yield the associated expected number of targets ${\hat{n}}_{t\left|t-1\right.}$ and ${\hat{n}}_{t}$.

According to the prediction step, the mean value of the predicted number of targets is

(49) $${\hat{n}}_{t\left|t-1\right.}={\hat{n}}_{t-1}p_{s,t}+\sum_{j=1}^{J_{\gamma ,t}}w_{\gamma ,t}^{j}$$

According to the update step, the mean value of the updated target number is

(50) $${\hat{n}}_{t}={\hat{n}}_{t\left|t-1\right.}\left(1-p_{d,t}\right)+\sum_{z\in Z_{t}}\sum_{j=1}^{J_{t\left|t-1\right.}}w_{t}^{j}\left(z\right)$$

(3) Pruning & Merging

The GP-PHD filter encounters the same computational challenges as the standard GM-PHD filter, especially the growth of the Gaussian components over time. To address this issue, an efficient pruning strategy is employed to reduce the number of Gaussian components passed to subsequent time steps [15]. The specific steps of the GP-PHD algorithm are described in Algorithm 1.
**Algorithm 1** The GP-PHD algorithm**Input:** ${\left\{w_{t-1}^{a},m_{t-1}^{a},P_{t-1}^{a}\right\}}_{a=1}^{J_{t-1}}$, $Z_{t}$, $T_{p}$, $T_{o}$ 1:Predict 2:(1) predict newborn targets 3:a=0 4:**for** $j=1:J_{\gamma ,t}$ **do** 5:   a=a+1 6:   $w_{t\left|t-1\right.}^{i}=w_{\gamma ,t}^{j}$, $m_{t\left|t-1\right.}^{i}=m_{\gamma ,t}^{j}$, $P_{t\left|t-1\right.}^{i}=P_{\gamma ,t}^{j}$ 7:**end for** 8:(2) predict existing targets 9:**for** $j=1:J_{t-1}$ **do**10:   a=a+111:   use (29)–(33) calculate the predictive parameters $m_{s,t\left|t-1\right.}^{j}$ and $P_{s,t\left|t-1\right.}^{j}$ for the birth targets12:**end for**13:$J_{t\left|t\right.-1}=i$14:Update15:**for** $j=1:J_{t\left|t\right.-1}$ **do**16:   $w_{t}^{a}=\left(1-p_{d,t}\right)w_{t\left|t-1\right.}^{a}$, $m_{t}^{a}=m_{t\left|t-1\right.}^{a}$, $P_{t}^{a}=P_{t\left|t-1\right.}^{a}$17:**end for**18:q=019:**for** $b=1:length(Z_{t})$ **do**20:   q=q+121:   **for** $j=1:J_{t\left|t\right.-1}$ **do**22:       $w_{t}^{j}=p_{d,t}w_{t\left|t-1\right.}^{j}q_{t}^{j}\left(z\right)$23:       use (36), (38)–(48) calculate the update parameters $m_{t\left|t\right.}^{j}$ and $P_{t\left|t\right.}^{j}$24:   **end for**25:   use (37) calculate the update parameters $w_{t}^{j}$26:**end for**27:$J_{t}=qJ_{t\left|t-1\right.}+J_{t\left|t-1\right.}$**Output:** ${\left\{w_{t}^{i},m_{t}^{i},P_{t}^{i}\right\}}_{i=1}^{J_{t}}$

## 5. Simulation Experiments

### 5.1. Performance Evaluation

To evaluate the effectiveness of the proposed GP-PHD filtering algorithm in this part, employ the Generalized Optimal Subpattern Assignment (GOSPA) distance [35], which is defined as

(51) $$d_{p}^{\left(c,\alpha \right)}\left(X,Y\right)≜{\left[\min_{\gamma \in \Gamma }\left(\sum_{\left(i,j\right)\in \gamma }d{\left(x_{i},y_{j}\right)}^{p}+\frac{c^{p}}{\alpha }\left(\left|X\right|+\left|Y\right|-\alpha \left|\gamma \right|\right)\right)\right]}^{\frac{1}{P}}$$

The parameters are assigned to c=50, p=2, α=2.

### 5.2. Simulation Results

(1) Scenario 1: For a two-dimensional surveillance region [−800,800]×[−800,800] m contains clutter and an unknown number of targets which evolve over time. Each target moves autonomously according to its motion model

(52) $$x_{t}=F_{CV/CT}x_{t-1}+\phi_{t}$$

(53) $$F_{CV}=\left[\begin{array}{cccc} 1 & \Delta & 0 & 0\\ 0 & 1 & 0 & 0\\ 0 & 0 & 1 & \Delta \\ 0 & 0 & 0 & 1 \end{array}\right]$$

(54) $$F_{CT}=\left[\begin{array}{cccc} 1 & \frac{\left(\sin\theta \right)}{\theta } & 0 & -\frac{\left(1-\cos\theta \right)}{\theta }\\ 0 & \cos\theta & 0 & -\sin\theta \\ 0 & \frac{\left(1-\cos\theta \right)}{\theta } & 1 & \frac{\left(\sin\theta \right)}{\theta }\\ 0 & \sin\theta & 0 & \cos\theta \end{array}\right]$$

with $\phi_{t}∼N\left(0,Q_{t}\right)$

(55) Qt=σ2Δ4Δ444Δ3Δ32200Δ3Δ322Δ20000Δ4Δ444Δ3Δ32200Δ3Δ322Δ3Δ322

where σ=0.1, Δ=1 s represents the sampling interval. Model 1 is a CV model (M1); Model 2 has a turn rate of θ=−9°/s and represents a left-turning model (M2); Model 3 is a right-turn model and the turn rate is θ=6°/s (M3). For each target, the survival probability and detection probability are $p_{s,t}=0.97$ and $p_{d,t}=0.95$, respectively. The observation consists of the orientation and distance

(56) $$z_{t}=\left[\begin{array}{c} \arctan\left(\frac{\zeta_{y}}{\zeta_{x}}\right)\\ \sqrt{\zeta_{x}^{2}+\zeta_{y}^{2}} \end{array}\right]+\varsigma_{t}$$

where $\varsigma_{t}∼N\left(0,R_{t}\right)$, $R_{t}=diag\left({\left[\sigma_{\theta }^{2},\sigma_{r}^{2}\right]}^{T}\right)$, σθ=2×ππ180180 rad/s, $\sigma_{r}=10$ m. The clutter model is modeled using a uniform Poisson model with a clutter rate $\lambda_{c}=10$. Additionally, a GM of the form is also utilized as the birth model of the target

(57) $$\gamma_{t}\left(x\right)=\sum_{i=1}^{5}w_{b}^{i}N\left(x;m_{b}^{i},P_{b}^{i}\right)$$

where $w_{b}^{i}=0.1$ and

$m_{b}^{1}={\left[\begin{array}{cccc} 50 & 0 & 250 & 0 \end{array}\right]}^{T}$, $m_{b}^{2}={\left[\begin{array}{cccc} -250 & 0 & -250 & 0 \end{array}\right]}^{T}$,
$m_{b}^{3}={\left[\begin{array}{cccc} -250 & 0 & 250 & 0 \end{array}\right]}^{T}$, $m_{b}^{4}={\left[\begin{array}{cccc} 250 & 0 & -250 & 0 \end{array}\right]}^{T}$,
$m_{b}^{5}={\left[\begin{array}{cccc} 0 & 0 & 150 & 0 \end{array}\right]}^{T}$, $P_{b}^{i}=diag\left({\left[200,100,200,100\right]}^{T}\right)$.

**Figure 2:**
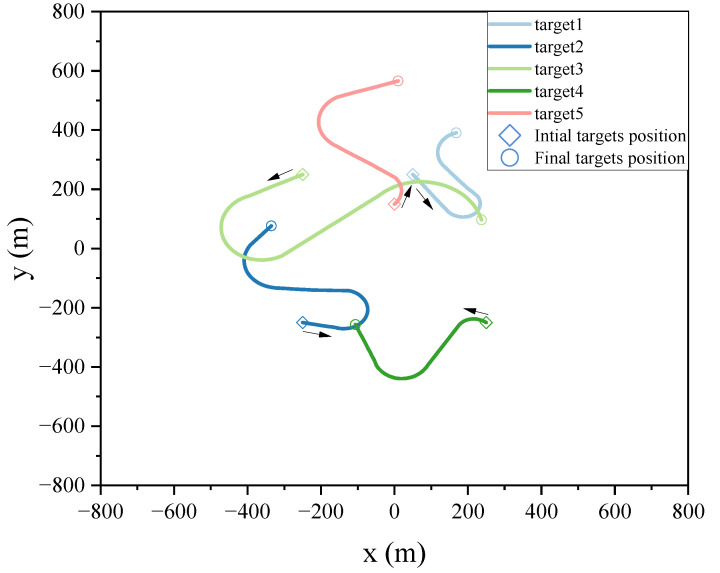
True trajectory of maneuvering targets.

The length of the training data $L_{1}=1000$, and the length of testing data $L_{2}=100$. The real trajectories used for training and testing are distinct, i.e., the training and testing data are from different datasets but follow the same motion model. For the targets’ motion process, the testing target moves in M2 at 20∼40 s, M3 at 60∼80 s, and M1 at other moments. Figure 2 displays the trajectory of the test targets. Furthermore, the efficacy of the proposed approach is evaluated by averaging 500 independent Monte Carlo (MC) experiments.

**Figure 3:**
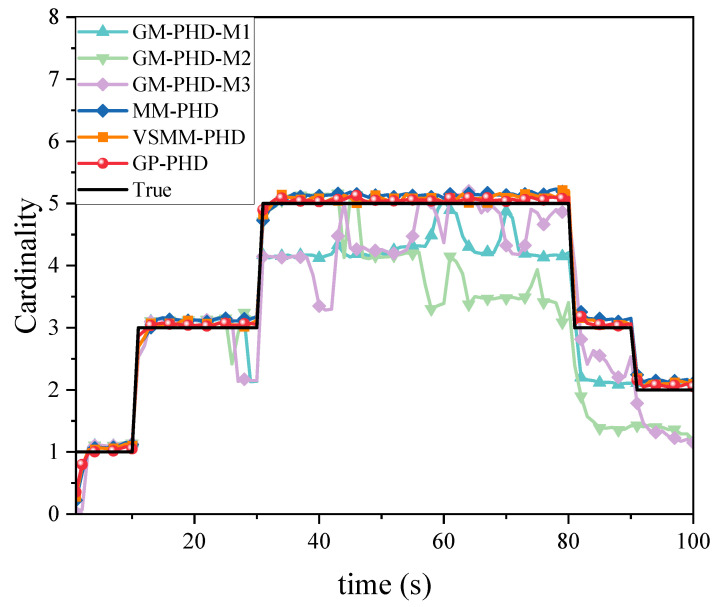
Cardinality estimation comparison under $p_{d}=0.95$.

**Figure 4:**
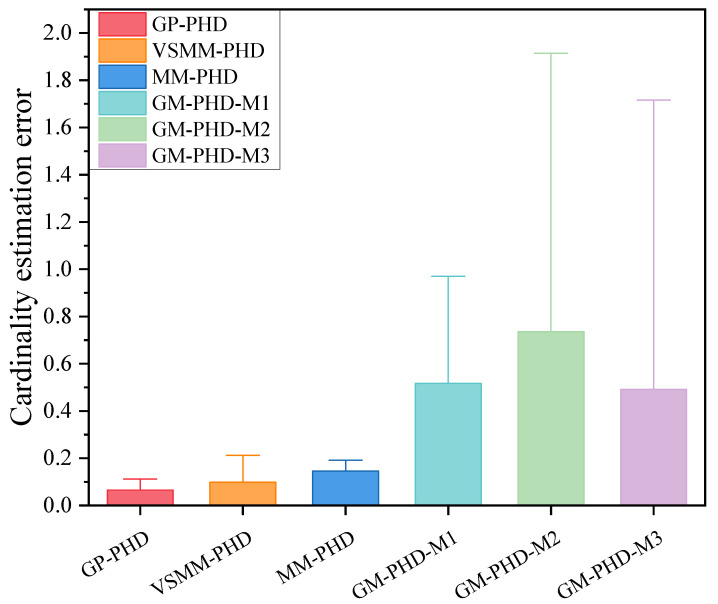
Cardinality estimation error comparison under $p_{d}=0.95$.

Figure 3 and Figure 4 illustrate the cardinality estimation and cardinality estimation error with detection probability $p_{d}=0.95$, respectively. The results in Figure 3 indicate that both the GP-PHD, VSMM-PHD, and MM-PHD filters outperform the single-model PHD filter in terms of performance and stability of cardinality estimation. When there is a significant model mismatch, the cardinality estimate error of the single model PHD filter increases observably and, therefore, cannot accurately estimate the actual number of targets in the environment. In contrast, the GP-PHD, VSMM-PHD, and MM-PHD filters show similar performance in MMTT cardinality estimation. A closer analysis reveals that the GP-PHD filter outperforms the others in target cardinality estimation. The histogram with error bars for cardinality estimation errors of several algorithms is shown in Figure 4, which is intended to visually and accurately present the mean value of cardinality estimation errors and their fluctuations of each algorithm so as to provide strong support for the comparison of different algorithms in terms of cardinality estimation accuracy. In this figure, the height of the histogram represents the mean value of the cardinality estimation error, while the error bars serve as a quantitative indicator of the fluctuation or uncertainty of the data, and the longer the error bars are, the greater the fluctuation or uncertainty of the data. After careful analysis, it can be clearly observed that the proposed GP-PHD algorithm performs the best in terms of the mean value of cardinality estimation error with the smallest mean value, which fully proves the excellent performance of the algorithm in the task of multi-maneuvering target cardinality estimation. Meanwhile, the VSMM-PHD and MM-PHD filters exhibit similar performance in cardinality estimation, but the VSMM-PHD filter shows a slight advantage in the mean value of cardinality estimation error. In contrast, the other single-model algorithms perform poorly in terms of both the mean cardinality estimation error and the range of fluctuation, which are large and fluctuate significantly, demonstrating significant shortcomings in cardinality estimation performance. This phenomenon further underscores the accuracy and stability of the GP-PHD algorithm for cardinality estimation of multi-maneuvering targets in complex environments.

**Figure 5:**
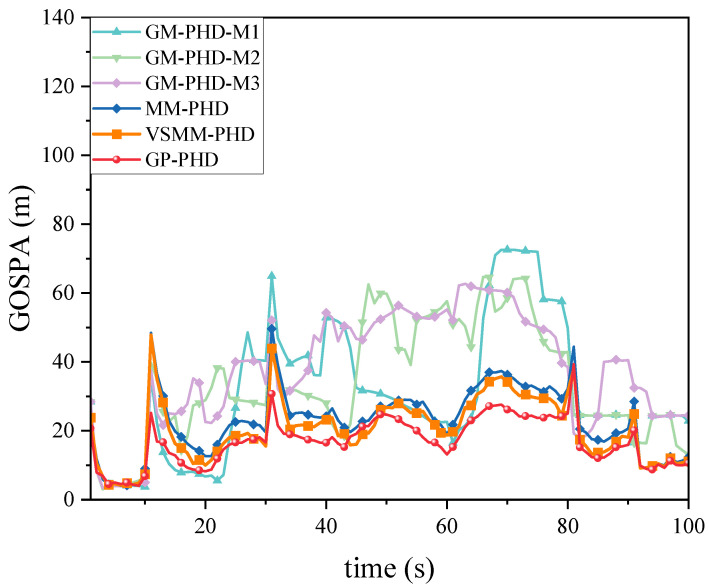
GOSPA distance under $p_{d}=0.95$.

**Figure 6:**
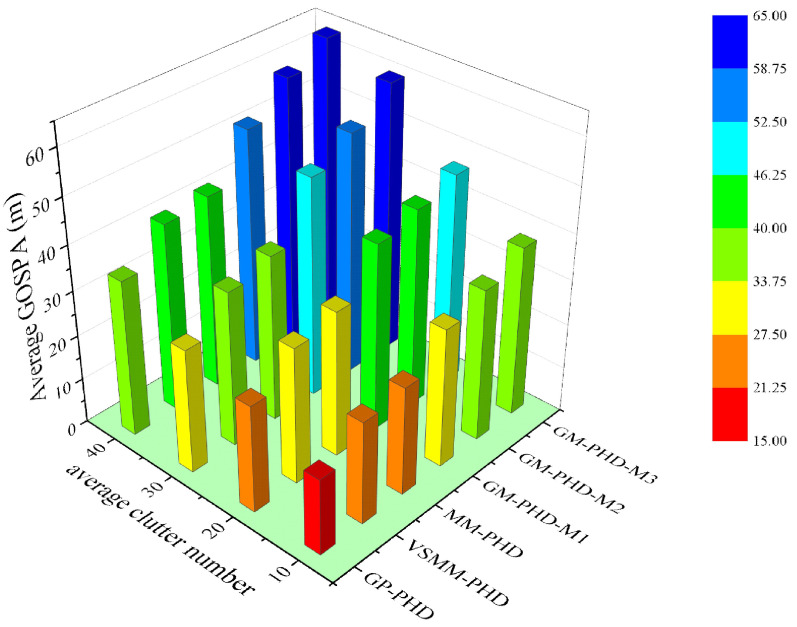
Average GOSPA distance under different clutter number under $p_{d}=0.95$.

Figure 5 and Figure 6 show the GOSPA distance with detection probability $p_{d}=0.95$, under various clutter conditions. Figure 5 demonstrates that the GP-PHD filter has an advantage over the VSMM-PHD, MM-PHD, and other single-model filters. By better adapting to changes in maneuvering target kinematics, the GP-PHD filter results in a smaller GOSPA distance. This is due to the GP’s ability to model the target’s dynamic properties flexibly, automatically learn the target’s motion models, adapt to different motion trajectories, and thus reduce the position estimation error. In addition, the precise modeling of the target motion can also effectively cope with the uncertainty of the target potential, thus reducing the occurrence of missed targets and false detections. This property plays a crucial role in reducing the GOSPA distance. Therefore, the GP-PHD filter outperforms other algorithms in terms of GOSPA distance. For instance, during the 40–60 s and 60–80 s, when the motion model of the maneuvering target changes, the GP-PHD filter maintains stable estimation performance with minimal degradation in accuracy. In contrast, the VSMM-PHD and MM-PHD filters do not perform as well as the GP-PHD filter because the multi-model approach generally suffers from model assumption limitations and model switching lags. These issues lead to increased errors in target location and cardinality estimation, thereby adversely affecting the GOSPA distance. Furthermore, when a single-model PHD filter is used for estimation, significant estimation errors are often observed due to the mismatch between the model and the actual target motion. Figure 6 illustrates the average GOSPA distance under varying clutter conditions. The average GOSPA distance for all algorithms tends to increase as the clutter density increases. However, the average GOSPA distance of the GP-PHD filter is less sensitive to the clutter density, maintaining the best estimation performance across all conditions. This further highlights the advantages of the GP-PHD filter in MMTT and its strong adaptability to complex environments.

**Figure 7:**
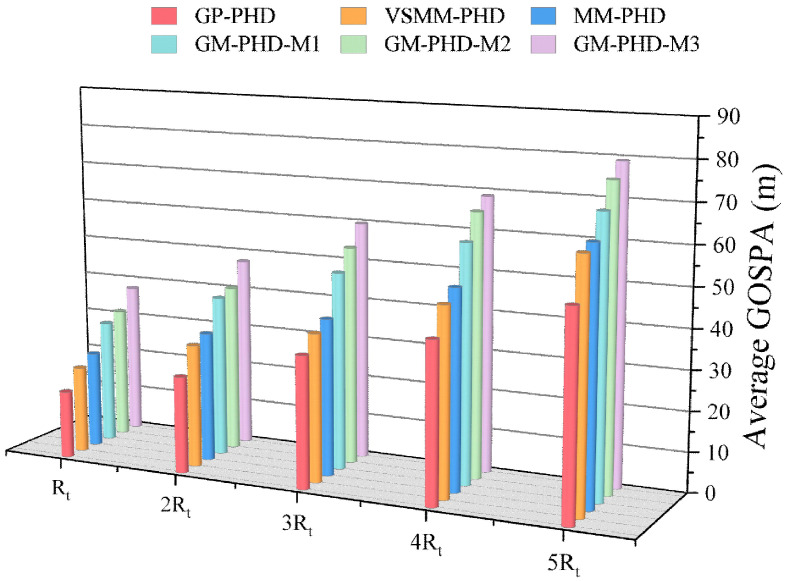
Average GOSPA distance under different $R_{t}$ under $p_{d}=0.95$.

To thoroughly assess the performance of the proposed algorithm in a low-signal-to-noise ratio (SNR) environment, Figure 7 demonstrates the average GOSPA distance of the algorithm under different settings of the measurement noise covariance. An increase in the measurement noise covariance matrix $R_{t}$, a key parameter affecting the SNR, leads to a reduction in SNR. It can be observed through Figure 7 that the GP-PHD filter exhibits the smallest GOSPA distance in each noise level test, highlighting its significant advantage in target tracking accuracy and robustness to noise interference. This advantage stems from the GP filter’s non-parametric modeling capability, which not only effectively learns the features of the target model but also adapts to the unknown characteristics of the noise covariance. Meanwhile, the VSMM-PHD and MM-PHD filters perform acceptably under initial low-noise conditions. Still, the GOSPA distance of these two filters increases rapidly with the growth in the measurement noise covariance, indicating a significant deficiency in their adaptability in high-noise environments. The performance degradation of the other single-model PHD filters is more significant in the presence of increased noise, underscoring the limitations of the single-model algorithm in terms of flexibility and estimation accuracy.

**Figure 8:**
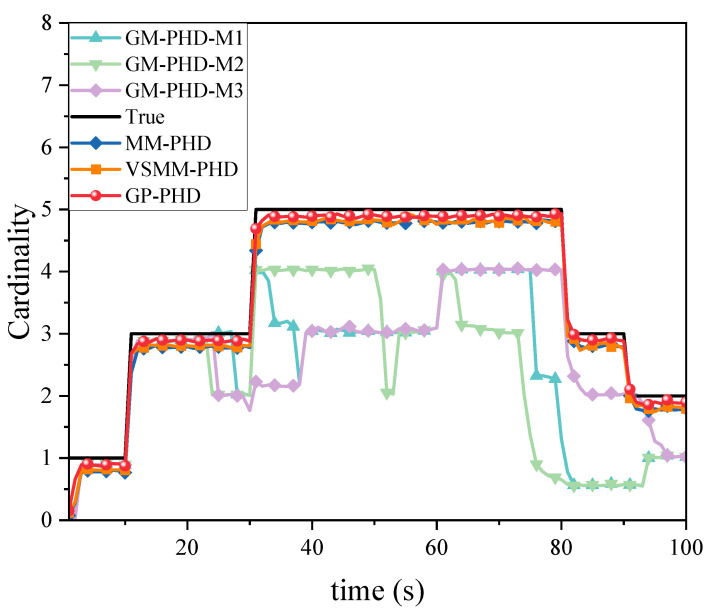
Cardinality estimation comparison under $p_{d}=0.7$.

**Figure 9:**
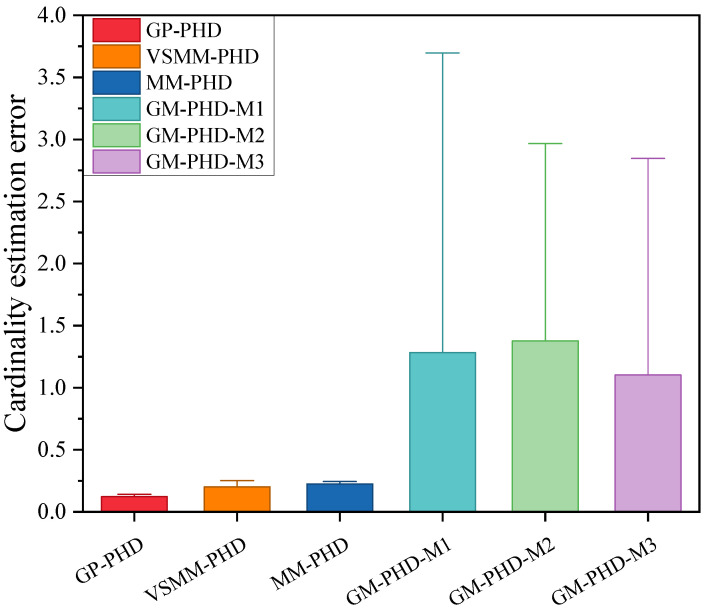
Cardinality estimation error comparison under $p_{d}=0.7$.

**Figure 10:**
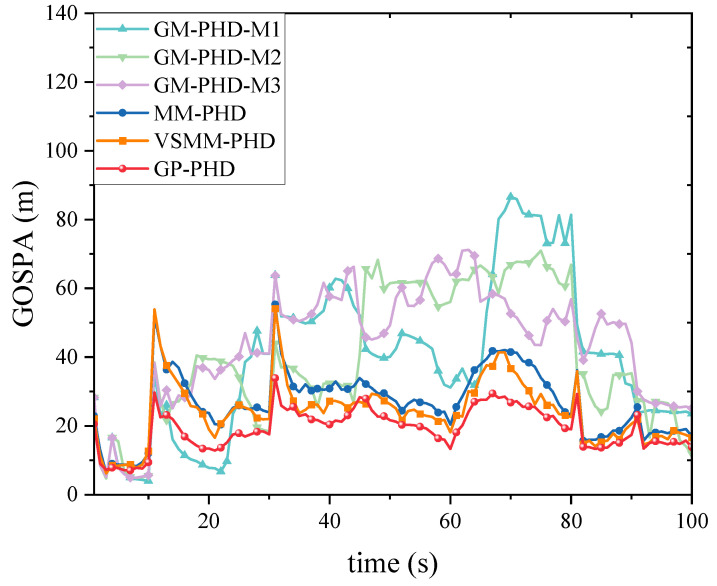
GOSPA distance under $p_{d}=0.7$.

Figure 8, Figure 9 and Figure 10 evaluate the tracking performance of different algorithms with a detection probability of 0.7. Figure 8 and Figure 9 show that a lower detection probability significantly affects the cardinality estimation of multi-maneuvering targets, with all algorithms exhibiting some bias. However, the cardinality estimation of the GP-PHD filter remains closer to the actual situation. In contrast, the VSMM-PHD and MM-PHD filters show more significant deviations, while the other single-model methods deviate even more. Figure 9 further illustrates this phenomenon using cardinality estimation error statistics. Despite the impact of low detection probability, the GP-PHD filter maintains better robustness in cardinality estimation and outperforms traditional MD algorithms. Figure 10 compares the GOSPA distance and shows that the proposed GP-PHD filter outperforms both MM-PHD and single-model PHD filters. This also highlights that the GP-PHD filter is beneficial in MMTT estimate. The GP-PHD filter demonstrates superior performance by maintaining a lower GOSPA distance even under challenging conditions with low detection probability.

Table 1 presents the average GOSPA distance of various filtering algorithms for 500 MC experiments at a detection probability of 0.7 under different clutter conditions. As the amount of clutter increases, the average GOSPA distance for all filters increases accordingly. However, the proposed GP-PHD filter exhibits a low average statistical error in these scenarios, highlighting its superiority in estimating multi-maneuvering target motion states when facing uncertain motion and observation models. In contrast, the MD MM-PHD filter performs slightly worse than the GP-PHD filter algorithm, while the other three single-model PHD filters perform poorly in low detection probability scenarios due to mismatched motion models. This difference shows that the GP-PHD filter maintains robust performance even under challenging conditions with low detection probability and high clutter rates.

(2) Scenario 2: A more complex MMTT environment is designed to further validate the effectiveness of the proposed approach. In this experimental setup, the maneuverability of the targets is significantly increased, imposing higher demands on the estimation performance of the MTT algorithms. The targets’ motion models still include M1, M2, and M3, but the turning rates of M2 and M3 have significantly changed with θ=−12 °/s and θ=12 °/s. This complex environment makes the trajectories of targets more diverse and uncertain, which poses greater challenges to the adaptability and robustness of tracking algorithms. Through this setup, the performance of the GP-PHD filter in highly dynamic and complex environments can be comprehensively evaluated and compared with other traditional MD algorithms. In addition, a GM of the form is employed, as well as the target birth model

(58) $$\gamma_{t}\left(x\right)=\sum_{i=1}^{5}w_{b}^{i}N\left(x;m_{b}^{i},P_{b}^{i}\right)$$

with $w_{b}^{i}=0.1$ and

$m_{b}^{1}={\left[\begin{array}{cccc} 50 & 0 & 250 & 0 \end{array}\right]}^{T}$, $m_{b}^{2}={\left[\begin{array}{cccc} -250 & 0 & -250 & 0 \end{array}\right]}^{T}$,
$m_{b}^{3}={\left[\begin{array}{cccc} -250 & 0 & 250 & 0 \end{array}\right]}^{T}$, $m_{b}^{4}={\left[\begin{array}{cccc} 250 & 0 & -250 & 0 \end{array}\right]}^{T}$,
$m_{b}^{5}={\left[\begin{array}{cccc} -100 & 0 & -100 & 0 \end{array}\right]}^{T}$.

**Figure 11:**
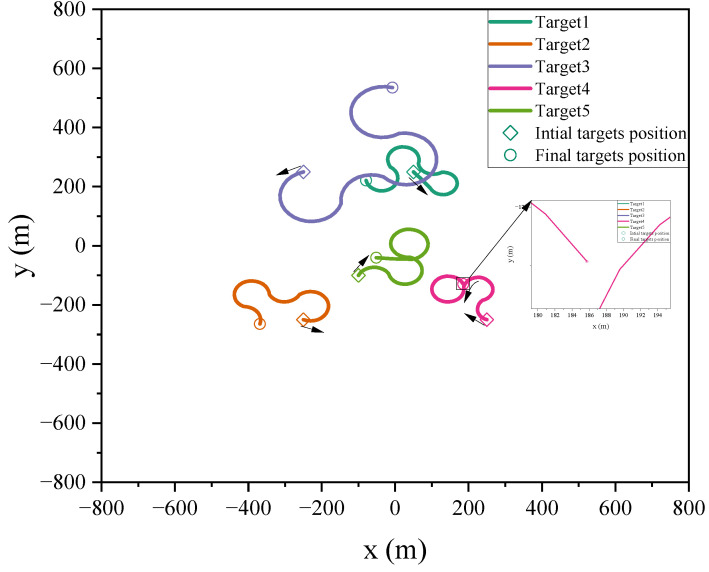
True trajectory of maneuvering targets.

The remaining of the multi-target motion and tracking environment parameters are set as in Scenario 1. The testing targets move in M2 during 10∼30 s and 41∼60 s, M3 during 31∼40 s and 61∼90 s, and M1 during the other intervals. The efficacy of the proposed approach is further validated through the aggregating 500 independent MC experiments. In Scenario 2, the actual trajectory used for testing is shown in Figure 11. As can be seen in the figure, the maneuverability of the targets has significantly increased due to changes in their turning rates. The intense maneuver introduces more significant uncertainty, which poses a more substantial challenge for tracking moving targets.

**Figure 12:**
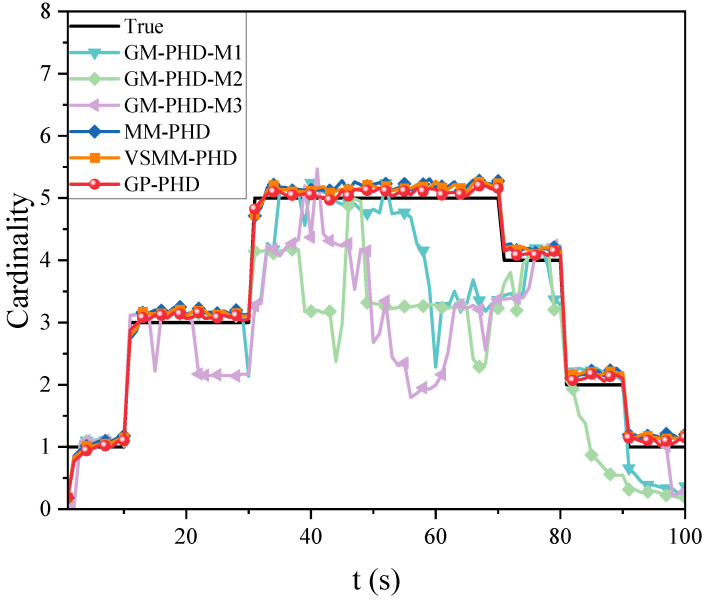
Cardinality estimation comparison under $p_{d}=0.95$.

Figure 12 compares the cardinality estimation for MMTT in a highly dynamic scenario. It is observed that the high maneuverability of the target movements significantly influences the cardinality estimation of multiple targets. The GP-PHD, VSMM-PHD, and MM-PHD filters exhibit varying degrees of deviation in their cardinality estimation. However, the GP-PHD filter, with its ability to learn motion models, better adapts to different maneuvering variations and outperforms both the VSMM-PHD and MM-PHD in multi-target cardinality estimation. Other single-model approaches generally fail to account for such maneuvering variations and, in most cases, do not accurately estimate the cardinality of multiple targets.

**Figure 13:**
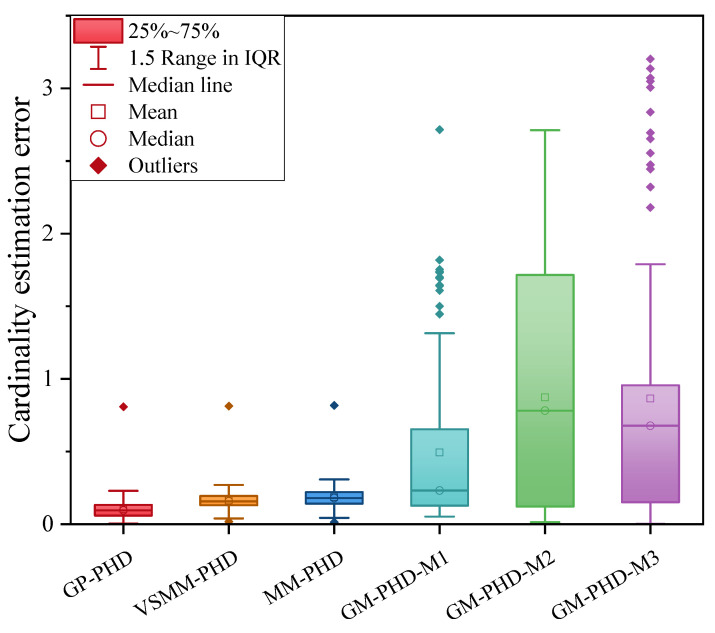
Cardinality estimateion error comparison under $p_{d}=0.95$.

Figure 13 further elucidates the differences between the algorithms using the cardinality estimation error statistics. The results indicate that although the cardinality estimation error statistics of the GP-PHD, VSMM-PHD, and MM-PHD filters exhibit similar performance, notable differences still exist. Compared to the VSMM-PHD and MM-PHD filters, the GP-PHD filter demonstrates smaller mean and median of the error statistics of cardinality estimation, highlighting its higher stability and accuracy in multi-target cardinality estimation. For the VSMM-PHD and MM-PHD filters, it is observed that there is no significant difference between the two in terms of cardinality estimation error, with the VSMM-PHD filter exhibiting a slight advantage. Other single-model filters exhibit issues such as scattered data, high variability, and numerous outliers, which render them inadequate for such a highly dynamic environment. The performance illustrated in Figure 12 and Figure 13 underscores the robustness and adaptability of the GP-PHD filter in tracking highly maneuverable targets. The GP-PHD filter’s ability to learn and adapt to different motion models ensures a more accurate and reliable cardinality estimate, even in challenging scenarios with significant target maneuverability.

**Figure 14:**
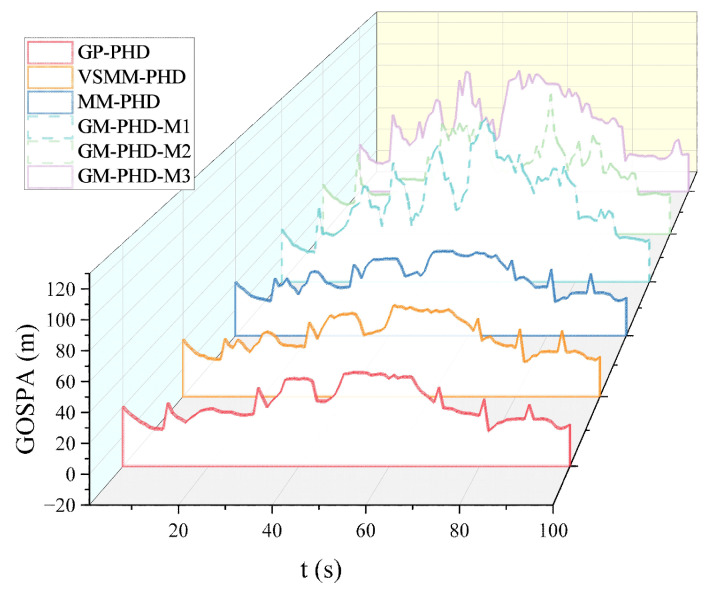
GOSPA distance under $p_{d}=0.95$.

**Figure 15:**
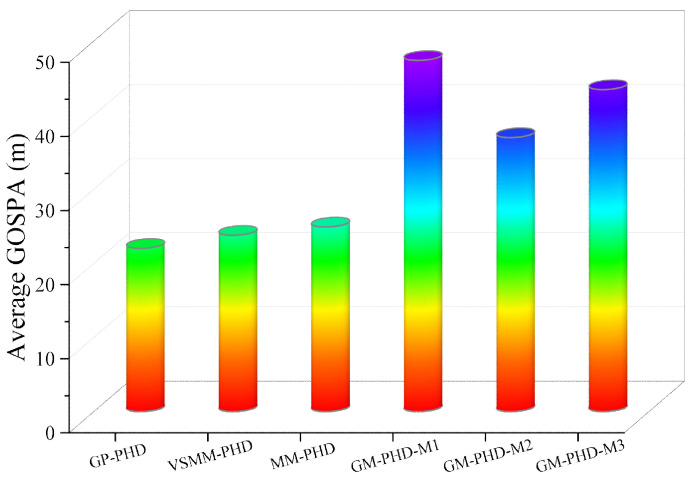
Average GOSPA distance under $p_{d}=0.95$.

Figure 14 and Figure 15 present the GOSPA distance and the average GOSPA distance for MMTT. Figure 14 shows that the GP-PHD, VSMM-PHD, and MM-PHD filters exhibit smaller GOSPA distance than other single-model filters, indicating higher accuracy in estimating target positions, missed detections, and false alarms. Variations in target states lead to fluctuations in GOSPA distance, as observed in periods such as 40∼50 s and 50∼70 s, where changes in target motion states and increased target counts result in significant increases in GOSPA distance. Notably, the GP-PHD filter shows a more stable GOSPA distance variation and is less sensitive to environmental changes than the other filters.

Figure 15 displays the average GOSPA distance, with the GP-PHD filter exhibiting the smallest average GOSPA distance, further confirming its superiority in MMTT. These results highlight the robustness and adaptability of the GP-PHD filter in complex scenarios. Compared to traditional methods, the GP-PHD filter can estimate MMTT states more accurately and achieve a smaller GOSPA distance, thereby underscoring its effectiveness. Overall, the GP-PHD filter maintains a smaller GOSPA distance even under significant changes in target motion, demonstrating its superiority in handling dynamic and complex environments. It can adapt to various target motion models while ensuring precise tracking, greatly enhancing the potential application of the GP-PHD filter.

Table 2 presents the average GOSPA distance for different detection probability conditions. The table shows that as detection probability decreases, the estimated performance of both GP-PHD and other filters shows a declining trend. However, the performance of the GP-PHD filter consistently outperforms that of VSMM-PHD, MM-PHD, and single-model PHD filters. This advantage is particularly important in real-world applications, where environmental factors can cause fluctuations in detection probability, making it essential to reliably and accurately track targets under diverse and challenging conditions. The GP-PHD filter maintains higher tracking accuracy even at low detection probabilities, indicating its adaptability and robustness in highly uncertain environments. In contrast, VSMM-PHD, MM-PHD, and single-model PHD filters exhibit noticeable performance degradation under low detection probability conditions and struggle to track multiple maneuvering targets reliably. This further underscores the advantage of the GP-PHD filter in MMTT applications, especially in dynamic and uncertain target motion and observation models.

**Figure 16:**
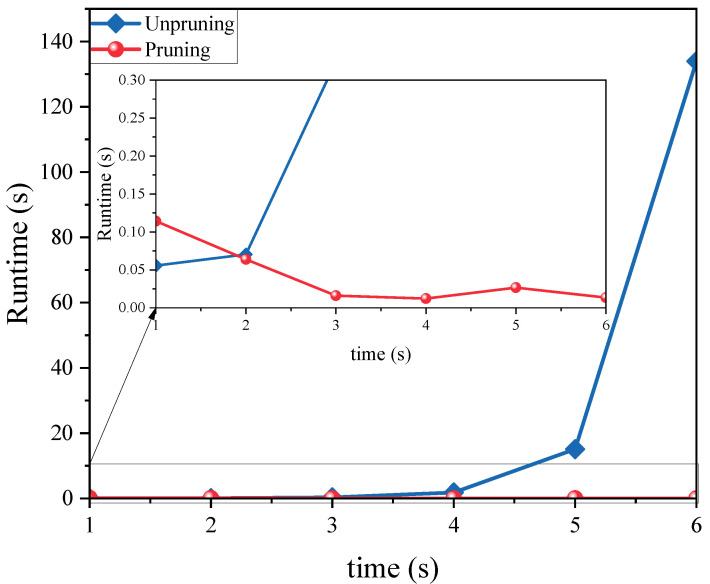
Runtime comparison.

Pruning plays a crucial role in the proposed algorithm, and it largely determines the computational efficiency of the algorithm. Figure 16 deeply analyzes the impact of pruning on the performance of the algorithm in practical applications by analyzing the execution time. Both cases are implemented in the MATLAB (2021b) environment on a computer equipped with a 3.9 GHz CPU (Inter Core i3-7100) (Santa Clara, CA, USA). From the comparison of the data in the figure, it is obvious that the algorithm with pruning algorithm maintains a stable and efficient performance at all time points. In contrast, the running time of the unpruned algorithm increases sharply with the increase in the number of Gaussian components. This trend significantly reduces the applicability of the algorithm in practical scenarios. Therefore, introducing the pruning step is of great significance in ensuring the real-time and practicality of the algorithm.

(3) Summary: Through a series of simulation experiments in different scenarios, the proposed GP-PHD filter demonstrates superior robustness when compared to the traditional tracking methods, and it effectively adapts to the complexity and uncertainty of the target motion in the tracking scenarios more effectively. This advantage is primarily reflected in the following aspects: (1) The GP-PHD filter can adaptively capture the dynamic behavior of the target without reliance on specific model assumptions, due to the modeling flexibility of GP. This characteristic makes the method particularly suitable for handling complex and variable target motion scenarios and can effectively address sudden maneuvers and nonlinear motion trajectories of the target. (2) The GP model has the ability to deal with similarities and differences in target motion, which makes the GP-PHD filter able to accurately distinguish and track the trajectories of different targets in complex scenarios when facing multi-target interactions. (3) The GP model can effectively deal with the uncertainty and noise in the observation, and the filter can still maintain excellent tracking performance even under conditions of low detection probability or serious clutter interference. Therefore, the GP-PHD filter shows its unique advantages and wide applicability in dealing with the challenges in the field of MMTT and offers an effective solution to the MMTT problem in complex environments.

## 6. Conclusions

This study proposes a model-free GP-PHD filter to effectively address the challenges of target motion and observation model uncertainty in MMTT. The filter leverages the GP to learn the unknown maneuvering targets’ motion and observation models and employs the ’cubature sampling’ method to create GM approximation of the posterior intensity for the next time step. Additionally, the study provides a concrete implementation of this filter utilizing the GM method. The experiments compare the performance of the GP-PHD filter with the VSMM-PHD, MM-PHD, and single-model GM-PHD filters. The results demonstrate that the GP-PHD filter exhibits robust adaptability in learning uncertain target motion and observation models, outperforming the VSMM, MM, and single-model methods. These advantages make the GP-PHD filter a preferred solution for MMTT. Its ability to learn and adapt to various target motion models ensures more accurate and reliable tracking in complex scenarios with highly maneuverable targets. In future research, applying the GP-PHD filter in multi-extended target tracking will be further explored for more challenging tracking tasks.

## Figures and Tables

**Table 1:** Average GOSPA distance statistics in different $\lambda_{c}$.

	$\lambda_{c}=10$	$\lambda_{c}=20$	$\lambda_{c}=30$	$\lambda_{c}=40$
GP-PHD	**18.81**	**28.82**	**39.19**	**53.52**
VSMM-PHD	24.26	36.43	47.57	64.93
MM-PHD	27.03	38.68	50.13	67.65
GM-PHD-M1	39.46	49.49	62.61	74.69
GM-PHD-M2	39.85	52.84	64.05	77.04
GM-PHD-M3	45.07	54.24	68.35	79.02

**Table 2:** Average GOSPA distance statistics in different $p_{d}$.

	$p_{d}=0.95$	$p_{d}=0.85$	$p_{d}=0.8$	$p_{d}=0.75$	$p_{d}=0.7$
GP-PHD	**22.03**	**23.87**	**25.63**	**27.72**	**30.64**
VSMM-PHD	23.15	24.04	26.49	28.93	32.75
MM-PHD	23.62	25.31	27.92	30.22	34.93
GM-PHD-M1	42.51	44.86	47.14	49.81	52.39
GM-PHD-M2	33.81	35.63	36.74	39.23	41.98
GM-PHD-M3	37.88	40.62	44.17	46.31	49.46

## Data Availability

The data are contained within this article.
